# Application of quantitative electroencephalography in predicting early cerebral ischemia in patients undergoing carotid endarterectomy

**DOI:** 10.3389/fneur.2023.1159788

**Published:** 2023-04-06

**Authors:** Guanxu Zhao, Guang Feng, Lei Zhao, Shuai Feng, Yi An, Cuicui Kong, Tianlong Wang

**Affiliations:** Department of Anesthesiology, Xuanwu Hospital, Capital Medical University, Beijing, China

**Keywords:** carotid endarterectomy, cerebral ischemia, quantitative electroencephalogram monitoring, transcranial doppler monitoring, intraoperative monitoring

## Abstract

**Background:**

Quantitative electroencephalography (QEEG) has emerged as a promising monitoring method in cerebral ischemia, but the feasibility of QEEG in intraoperative cerebral perfusion-related ischemia monitoring is still uncertain. The purpose of this study was to investigate the value of QEEG monitoring in Carotid Endarterectomy (CEA) and the thresholds for intraoperative cerebral perfusion-related ischemia monitoring.

**Methods:**

Sixty-three patients who underwent carotid endarterectomy with continuous Transcranial Doppler ultrasound (TCD) monitoring and QEEG monitoring at Xuanwu Hospital Capital Medical University from January 2021 to August 2021 were enrolled in this study. Each patient received total intravenous anesthesia. Middle cerebral artery blood flow velocity (V-MCA) was obtained by TCD. Relative alpha percentage (RA) and alpha-delta ratio (ADR) were obtained by QEEG monitoring. Patients were divided into ischemic and non-ischemic groups using a decline of more than 50% in the V-MCA monitored by TCD as the gold standard.

**Results:**

Of the 63 patients, twenty patients were divided into the ischemic group, and forty-three patients into the non-ischemic group. Ipsilateral post-clamp RA and ADR values of QEEG were decreased for all patients in the ischemic group. The optimal threshold for RA and ADR to predict cerebral ischemia was a 14% decrease from baseline (sensitivity 90.0%, specificity 90.7%, Kappa value 0.786), a 21% decrease from baseline (sensitivity 85.0%, specificity 81.4%, Kappa value 0.622), respectively, indicated by TCD monitoring.

**Conclusions:**

Our study demonstrated that QEEG is a promising monitoring method undergoing CEA under general anesthesia and has good consistency with TCD.

## Introduction

Worldwide, ischemic stroke accounts for about 68% and hemorrhagic stroke about 32%, while in China, the proportion of ischemic stroke is even more significant with 84%. Stroke is the second leading cause of death worldwide and the first leading cause of death in China (1–4). Stroke imposes a heavy burden on patients, families, and society. Therefore, prevention and treatment of stroke are of great importance.

About 10–20% of stroke is caused by carotid artery diseases, so timely intervention is crucial for the secondary or even primary prevention of stroke. Among the many factors leading to stroke, the degree of carotid artery stenosis is the most important determinant. Carotid endarterectomy (CEA) is a surgical method for reconstructing carotid blood flow and reducing the risk of stroke by removing calcified plaques inside vessels. Previous studies have confirmed that CEA has high benefits for patients with non-disabling stroke and severe carotid artery stenosis (70–99%), as well as patients with recent hemispheric and retina-related transient ischemic attack (TIA) (5–8). Although there are benefits for patients with severe stenosis, CEA requires temporary blocking of blood flow in one side of the carotid artery, which will lead to a significant reduction of cerebral blood flow at the clamping side of the hemisphere during CEA, and even ischemic stroke and other neurological adverse events. Previous studies have shown that 30 days perioperative stroke rates after CEA range between 2 and 6% (5, 9, 10). These make the benefits of patients dependent on the perioperative safety of CEA. In order to reduce the incidence of neurological adverse events during CEA and maximize the benefit of patients, intraoperative cerebral perfusion-related ischemia monitoring is essential. Intraoperative monitoring (IOM) can guide the surgeon to optimize surgical strategy and provide recommendations for shunting.

Many centers have used transcranial doppler (TCD) for IOM due to its convenient and non-invasive characteristics. However, TCD monitored the brain region with the middle cerebral artery as the blood supply and lack of monitoring in other areas. Besides, some patients don't have the appropriate temporal insonation window to be monitored with TCD. In recent years, Quantitative EEG (QEEG) has been attracting more and more attention as a new monitoring method (11, 12). QEEG uses Fourier transforms to quantify the raw EEG based on its amplitude, power, frequency, and rhythmicity to show numerical values, ratios or percentages, and a graphical display array or trend. It is now possible to calculate the ratio or percentage of α, β, θ, and δ power in a particular frequency band, such as relative alpha percentage (RA) and alpha-delta ratio (ADR), and these values can be compared between different regions, such as electrode pair channels. RA is the power of α wave as a percentage of power in 1–20 Hz band. ADR is the ratio of α wave power to δ wave power. Compared with raw EEG, one advantage of QEEG is that it allows for a more visual display of cerebral perfusion monitoring, another is that QEEG can monitor the whole brain perfusion, rather than only partial brain areas as TCD and cerebral oxygenation do. Laman et al. analyzed the value of QEEG parameters such as spectral edge frequency (SEF) and relative band power in predicting shunt in CEA. Kamitaki et al. evaluated the value of QEEG according to the responses of different QEEG parameters after carotid artery clamping during CEA under general anesthesia. Both of them obtained good results (13, 14). However, the two studies were retrospective, using EEG data from a database, and in Kamitaki's study, the grouping of patients is a subjective judgment by EEG physicians. At present, no specific threshold for QEEG changes in CEA has been proposed and there is no study on the combination of QEEG and TCD for cerebral perfusion during CEA.

The aim of the present study was to use a combination of QEEG and TCD to evaluate the feasibility of the new QEEG monitoring to monitor cerebral perfusion status during CEA, reducing the incidence of perioperative cerebral ischemia events.

## Materials and methods

### Participants

This prospective and observational study was conducted in Xuanwu Hospital Capital Medical University from January 2021 to August 2021. The study was approved by the institutional ethics board of Xuanwu Hospital Capital Medical University (approval [2020] No. 121). The study was registered with ClinicalTrials.gov (ChiCTR2000035330). All patients were informed and signed written informed consent before inclusion in the study. The inclusion criteria included: (1) Aged between 18 and 75 years old. (2) American Society of Anesthesiologists (ASA) grade ≤ III. New York Heart Association (NYHA) grade ≤ II. (3) Among patients undergoing CEA surgery for the first time, symptomatic patients who were diagnosed with internal carotid artery stenosis >50% or asymptomatic patients who were diagnosed with internal carotid artery stenosis >70% by preoperative digital subtraction angiography (DSA).

The exclusion criteria included: (1) Patients who refused to accept the study. (2) Emergency surgery. (3) ASA grade ≥ IV, NYHA grade ≥ III. (4) Epilepsy and other diseases affecting EEG monitoring. (5) Unable to perform transcranial Doppler ultrasound monitoring due to lack of appropriate temporal window.

### EEG monitoring and TCD monitoring

All patients received continuous EEG and TCD monitoring simultaneously after entering the operating room. TCD is the reference standard.

TCD was monitored using an ultrasonic Transcranial Doppler blood flow analyzer (Shenzhen Deli Kai Electronics Co., LTD., China). The ultrasound probe was placed in the temporal window above the zygomatic arch on both sides and operated by vascular ultrasound physicians to detect the blood flow velocity of the middle cerebral artery (V-MCA).

EEG was recorded using a trackit-24/0 dynamic EEG polysomnography recording box (G&B Electronic Designs Ltd., UK). EEG electrode cap was a disc-shaped gel electrode, a total of 18. Fp1, Fp2, F3, F4, F7, F8, C3, C4, P3, P4, T3, T4, T5, T6, O1, O2, GND, and Ref were placed on the scalp according to the international standard 10–20 system. The iEEG system software (Lifelines Ltd., UK) was used to automatically analyze the EEG data in real-time and obtain the necessary observation data: RA and ADR. The paper moving speed of EEG was 30 mm/s, the high-frequency filter was 35 Hz, and the low-frequency filter was 1.0 Hz.

All patients were divided into ischemic and non-ischemic groups according to whether the V-MCA decreased < 50% of baseline (15), and then compare the QEEG values between two groups.

### Anesthesia procedure

No preoperative anxiolytic medication was administered. Intraoperative monitoring included the ASA mandatory monitoring, Bispectral Index (BIS), and end-tidal gas monitoring. All patients received total intravenous anesthesia. Etomidate (0.2 mg/kg), rocuronium (0.6 mg/kg), and sufentanil (0.3 μg/kg) were used for induction. Maintenance of anesthesia is with propofol (3 to 6 mg·kg^−1^·h^−1^) and remifentanil (0.2 to 0.4 μg·kg^−1^·min^−1^). There were no limitations for the use of muscle relaxant and vasoactive medications.

During the operation, blood pressure was maintained within the range of ± 20% of the baseline, nasopharyngeal temperature was between 36 to 37.3°C, end-tidal pressure of carbon dioxide was between 30 to 40 mmHg. The BIS value was maintained between 40 to 60 by adjusting propofol dosages.

### Data collection

Four time points were selected. After endotracheal intubation was completed and blood pressure stabilized, we adjusted the blood pressure close to the preoperative resting state blood pressure. The blood pressure and TCD values at this time were set as baseline (T_B_). The time after carotid artery clamping for 30 s was recorded as T_1_, the time after carotid artery clamping for 1 min was recorded as T_2_, the time after carotid artery clamping for 2 min was recorded as T_3_. RA and ADR values combined with TCD values were recorded at each point of time, and the percentage change of each measured value compared to the baseline was calculated to facilitate further statistical analysis.

After the operation, the neurophysical examination was performed.

### Statistical analysis

The sample size was calculated using Power Analysis and Sample Size (PASS) software (version 15.0; NCSS Statistical Software, East Kaysville, UT, USA) to detect an area under the receiver operating characteristic curve (AUROC) of 0.8 given a null hypothesis of an AUROC of 0.5. According to the pre-test results, the rate of the V-MCA decreased < 50% of baseline was about 30%. We determined that a minimum of 55 patients (including 17 ischemia and 38 non-ischemia patients) were needed to obtain a study power of 90% with an α error of 0.05. Patients with missing data were not included in the current study.

Statistical Software SPSS 23.0 (IBM Corp, USA) and Medcalc 16.8.4 (Medcalc Software Ltd., Belgium) were used and significant differences were considered if P < 0.05. Measurement data were expressed as means ± standard deviation ($\bar{X}$±SD) description (normal distribution) or median (25th and 75th percentile) description (non-normal distribution). Enumeration data expressed as percentages. The independent sample *T*-test (normal distribution) or Mann-Whitney U test (non-normal distribution) was used for comparison of measurement data between groups. The paired-sample *T*-test (normal distribution) or Wilcoxon signed-rank test (non-normal distribution) were used for intragroup comparison of measurement data. Receiver operating characteristic (ROC) curves were used to verify the cut-off point of QEEG indicators for predicting cerebral ischemia. The sensitivity and specificity of RA and ADR, as well as their consistency with TCD monitoring, were calculated using TCD monitoring values as the standard.

## Results

### General characteristics of patients

From January 2021 to August 2021, 152 patients intending to have a CEA were assessed for eligibility, of whom 113 patients met inclusion criteria and were considered potential study candidates. Among these, 30 patients declined participation and 16 patients were not approached by the researchers. Therefore, a total of 67 patients were enrolled in this study. After excluding four patients with poor EEG data, a total of 63 patients were analyzed. The patients were divided into ischemic and non-ischemic groups for statistical analysis based on the criterion that the blood flow velocity of the middle cerebral artery monitored by TCD decreased to < 50% of baseline. As shown in Table 1, there was no statistical significance in the general information, surgical side, preoperative symptoms, contralateral severe carotid artery stenosis, and previous history of the two groups (*P* > 0.05).

**Table 1:** Patients demographics and characteristics.

	**Ischemic group**	**Non-ischemic group**	***P*-value**
**Patient characteristics**			
Number of patients	20	43	
Gender	Male (100%)	Male (100%)	
Mean age (±SD)	64.25 (±7.69)	63.23 (±7.50)	0.62
**Surgical information**			
Side of CEA (%)			0.09
Left	9 (45%)	29 (67.4%)	
Right	11 (55%)	14 (32.6%)	
Surgery for symptomatic or asymptomatic (%)			0.42
Symptomatic	17 (85%)	31 (72.1%)	
Asymptomatic	3 (15%)	12 (27.9%)	
Severe stenosis of contralateral ICA (%)			0.57
Yes	7 (40%)	12 (27.9%)	
No	13 (60%)	31 (72.1%)	
**Complications**			
Hypertension (%)	18 (90%)	33 (76.7%)	0.37
Coronary heart disease (%)	7 (35%)	11 (25.6%)	0.44
Diabetes mellitus (%)	8 (40%)	16 (37.2%)	0.83
Hyperlipidemia (%)	13 (65%)	35 (81.4%)	0.27
Smoking history (%)	16 (80%)	32 (74.4%)	0.87
**NIHSS**			0.16
0	16 (80%)	39 (90.6%)	
1	4 (20%)	2 (4.7%)	
2	0	2 (4.7%)	
**mRS**			0.63
0	15 (75%)	36 (83.7%)	
1	5 (25%)	7 (16.3%)	

### The effect of intraoperative cerebral hypoperfusion monitored by QEEG and its consistency with TCD

Twenty patients had cerebral hypoperfusion after clamping the internal carotid artery, with v-MCA values decreasing by more than 50% from baseline. Intraoperative shunt surgery was decided by the surgeon in 7 cases. According to the ROC curve, the distribution of the optimal cut-off point for RA to monitor cerebral hypoperfusion decreased by 11%−16% compared with baseline, and the Area Under ROC curve (AUC) ranged from 0.911–0.970 (*P* < 0.001) (Table 2). The distribution of optimal cut-off points for ADR monitoring cerebral hypoperfusion decreased by 20%−33% from baseline, and AUC ranged from 0.809 to 0.895 (*P* < 0.001) (Table 3).

**Table 2:** RA comparison of different leads at different time points on the surgical side undergoing CEA.

**QEEG parameters**	**T_1_**	**T_2_**	**T_3_**
**RA1**			
Cut-off	−14%	−14%	−16%
AUC	0.948	0.941	0.938
95%CI	0.897~0.999	0.884~0.999	0.881~0.996
*P*	< 0.001	< 0.001	< 0.001
**RA2**			
Cut-off	−13%	−11%	−11%
AUC	0.949	0.930	0.926
95%CI	0.900~0.998	0.870~0.989	0.864~0.998
*P*	< 0.001	< 0.001	< 0.001
**RA3**			
Cut-off	−14%	−11%	−13%
AUC	0.928	0.952	0.928
95%CI	0.865~0.992	0.905~0.998	0.865~0.992
*P*	< 0.001	< 0.001	< 0.001
**RA4**			
Cut-off	−15%	−15%	16%
AUC	0.917	0.939	0.911
95%CI	0.833~1.000	0.878~1.000	0.839~0.983
*P*	< 0.001	< 0.001	< 0.001
**RA5**			
Cut-off	−11%	−12%	−12%
AUC	0.959	0.970	0.962
95%CI	0.917~1.000	0.935~1.000	0.921~1.000
*P*	< 0.001	< 0.001	< 0.001
**RA6**			
Cut-off	−11%	−16%	−12%
AUC	0.947	0.934	0.938
95%CI	0.896~0.997	0.876~0.992	0.883~0.993
*P*	< 0.001	< 0.001	< 0.001
**RA7**			
Cut-off	−13%	−14%	−13%
AUC	0.955	0.966	0.963
95%CI	0.911~1.000	0.929~1.000	0.925~1.000
*P*	< 0.001	< 0.001	< 0.001
**RA8**			
Cut-off	−12%	−13%	−11%
AUC	0.938	0.951	0.950
95%CI	0.879~0.997	0.898~1.000	0.900~1.000
*P*	< 0.001	< 0.001	< 0.001
RA1: Fp1-F3/Fp2-F4	RA5: C3-P3/C4-P4	T_1_: After clamping 30 s	
RA2: F3-C3/F4-C3	RA6: P3-O1/P4-O2	T_2_: After clamping 1 min	
RA3: Fp1-F7/Fp2-F8	RA7: T3-T5/T4-T6	T_3_: After clamping 2 min	
RA4: F7-T3/F8-T4	RA8: T5-O1/T6-O2		

Negative numbers indicate a decrease from baseline.

**Table 3:** ADR comparison of different leads at different time points on the surgical side undergoing CEA.

**QEEG parameters**	**T_1_**	**T_2_**	**T_3_**
**ADR1**			
Cut-off	−29%	−24%	−22%
AUC	0.849	0.823	0.866
95%CI	0.743–0.956	0.695–0.951	0.761–0.972
*P*	< 0.001	< 0.001	< 0.001
**ADR2**			
Cut-off	−29%	−26%	−33%
AUC	0.809	0.880	0.872
95%CI	0.672–0.945	0.789–0.971	0.771–0.973
*P*	< 0.001	< 0.001	< 0.001
**ADR3**			
Cut-off	−33%	−30%	−23%
AUC	0.888	0.853	0.869
95%CI	0.799–0.977	0.742–0.964	0.773–0.964
*P*	< 0.001	< 0.001	< 0.001
**ADR4**			
Cut-off	−21%	−25%	−23%
AUC	0.861	0.851	0.850
95%CI	0.747–0.975	0.729–0.972	0.737–0.963
*P*	< 0.001	< 0.001	< 0.001
**ADR5**			
Cut-off	−21%	−20%	−27%
AUC	0.869	0.834	0.874
95%CI	0.763–0.974	0.701–0.966	0.768–0.980
*P*	< 0.001	< 0.001	< 0.001
**ADR6**			
Cut-off	−30%	−30%	−36%
AUC	0.895	0.845	0.847
95%CI	0.808–0.983	0.718–0.972	0.716–0.918
*P*	< 0.001	< 0.001	< 0.001
**ADR7**			
Cut-off	−30%	−23%	−30%
AUC	0.885	0.831	0.851
95%CI	0.778–0.993	0.684–0.977	0.709–0.992
*P*	< 0.001	< 0.001	< 0.001
**ADR8**			
Cut-off	−31%	−31%	−31%
AUC	0.860	0.860	0.835
95%CI	0.734–0.987	0.734–0.987	0.702–0.968
*P*	< 0.001	< 0.001	< 0.001
ADR1: Fp1-F3/Fp2-F4	ADR5: C3-P3/C4-P4	T_1_: After clamping 30 s	
ADR2: F3-C3/F4-C3	ADR6: P3-O1/P4-O2	T_2_: After clamping 1 min	
ADR3: Fp1-F7/Fp2-F8	ADR7: T3-T5/T4-T6	T_3_: After clamping 2 min	
ADR4: F7-T3/F8-T4	ADR8: T5-O1/T6-O2		

Negative numbers indicate a decrease from baseline.

According to the cut-off point values of different electrode pairs, the groups were regrouped with TCD monitoring results to make a four-grid table to calculate the sensitivity, specificity, positive predictive value, negative predictive value, and Kappa value of RA and ADR monitoring cerebral hypoperfusion. The sensitivity, specificity, positive predictive value, negative predictive value, and Kappa value of RA of different electrode channels for predicting cerebral ischemia at different time points ranged from 75–90%, 76.7–93%, 64.3–81.8%, 88.6–95.7%, and 0.583–0.786. The sensitivity, specificity, positive predictive value, negative predictive value, and Kappa value of ADR of different electrode channels at different time points for predicting cerebral ischemia ranged from 65–85%, 81.4–97.7%, 65.2–93.3, 85.1–92.1%, 0.542–0.733. The values are shown in Tables 4, 5.

**Table 4:** The accuracy of RA evaluation at different time points of different leads on the surgical side undergoing CEA according to the cut-off point value.

**QEEG parameters**	**T_1_**	**T_2_**	**T_3_**
**RA1**			
Sensitivity	90.0%	80.0%	85.0%
Specificity	90.7%	90.7%	90.7%
Positive predictive value	81.8%	80.0%	81.0%
Negative predictive value	95.7%	90.7	92.9%
Kappa value	0.786	0.707	0.747
*P*	< 0.001	< 0.001	< 0.001
**RA2**			
Sensitivity	80.0%	90.0%	80.0%
Specificity	88.4%	76.7%	83.7%
Positive predictive value	76.2%	64.3%	69.6%
Negative predictive value	90.5%	94.3%	90.0%
Kappa value	0.675	0.603	0.613
*P*	< 0.001	< 0.001	< 0.001
**RA3**			
Sensitivity	90.0%	85.0%	85.0%
Specificity	90.7%	90.7%	86.0%
Positive predictive value	81.8%	81.0%	73.9%
Negative predictive value	95.1%	92.9%	92.5%
Kappa value	0.786	0.747	0.683
*P*	< 0.001	< 0.001	< 0.001
**RA4**			
Sensitivity	85.0%	80.0%	80.0%
Specificity	90.7%	93.0%	81.4%
Positive predictive value	81.0%	84.2%	66.7%
Negative predictive value	92.9%	90.9%	89.7%
Kappa value	0.747	0.740	0.583
*P*	< 0.001	< 0.001	< 0.001
**RA5**			
Sensitivity	90.0%	90.0%	90.0%
Specificity	86.0%	90.7%	90.7%
Positive predictive value	75.0%	81.8%	81.8%
Negative predictive value	94.9%	95.1%	95.1%
Kappa value	0.722	0.786	0.786
*P*	< 0.001	< 0.001	< 0.001
**RA6**			
Sensitivity	80.0%	75.0%	80.0%
Specificity	81.4%	90.7%	88.4%
Positive predictive value	66.7%	78.9%	76.2%
Negative predictive value	89.7%	88.6%	90.5%
Kappa value	0.583	0.747	0.675
*P*	< 0.001	< 0.001	< 0.001
**RA7**			
Sensitivity	90.0%	90.0%	80.0%
Specificity	88.4%	88.4%	88.4%
Positive predictive value	78.3%	78.3%	76.2%
Negative predictive value	95.0%	95.0%	90.5%
Kappa value	0.753	0.753	0.675
*P*	< 0.001	< 0.001	< 0.001
**RA8**			
Sensitivity	80.0%	85.0%	90.0%
Specificity	86.0%	90.7%	90.7%
Positive predictive value	72.7%	81.0%	81.8%
Negative predictive value	90.2%	92.9%	95.7%
Kappa value	0.643	0.747	0.786
*P*	< 0.001	< 0.001	< 0.001
RA1: Fp1-F3/Fp2-F4	RA5: C3-P3/C4-P4	T_1_: After clamping 30 s	
RA2: F3-C3/F4-C3	RA6: P3-O1/P4-O2	T_2_: After clamping 1 min	
RA3: Fp1-F7/Fp2-F8	RA7: T3-T5/T4-T6	T_3_: After clamping 2 min	
RA4: F7-T3/F8-T4	RA8: T5-O1/T6-O2		

**Table 5:** The accuracy of ADR evaluation at different time points of different leads on the surgical side undergoing CEA according to the cut-off point value.

**QEEG parameters**	**T_1_**	**T_2_**	**T_3_**
**ADR1**			
Sensitivity	75.0%	75.0%	80.0%
Specificity	81.4%	83.7%	86.0%
Positive predictive value	65.2%	68.2%	72.7%
Negative predictive value	87.5%	87.8%	90.2%
Kappa value	0.542	0.572	0.643
*P*	< 0.001	< 0.001	< 0.001
**ADR2**			
Sensitivity	75.0%	75.0%	65.0%
Specificity	88.4%	86.0%	93.0%
Positive predictive value	75.0%	71.4%	81.3%
Negative predictive value	88.4%	88.1%	85.1%
Kappa value	0.634	0.602	0.613
*P*	< 0.001	< 0.001	< 0.001
**ADR3**			
Sensitivity	70.0%	70.0%	75.0%
Specificity	95.3%	88.4%	83.7%
Positive predictive value	87.5%	73.7%	68.2%
Negative predictive value	87.2%	86.4%	87.8%
Kappa value	0.690	0.592	0.572
*P*	< 0.001	< 0.001	< 0.001
**ADR4**			
Sensitivity	85.0%	80.0%	80.0%
Specificity	81.4%	88.4%	81.4%
Positive predictive value	68.0%	76.2%	66.7%
Negative predictive value	92.1%	90.5%	89.7%
Kappa value	0.622	0.675	0.583
*P*	< 0.001	< 0.001	< 0.001
**ADR5**			
Sensitivity	80.0%	75.0%	75.0%
Specificity	86.0%	86.0%	88.4%
Positive predictive value	72.7%	71.4%	75.0%
Negative predictive value	90.2%	88.1%	88.4%
Kappa value	0.643	0.602	0.634
*P*	< 0.001	< 0.001	< 0.001
**ADR6**			
Sensitivity	70.0%	75.0%	70.0%
Specificity	93.0%	90.7%	97.7%
Positive predictive value	82.4%	78.9%	93.3%
Negative predictive value	87.0%	88.6%	87.5%
Kappa value	0.657	0.666	0.725
*P*	< 0.001	< 0.001	< 0.001
**ADR7**			
Sensitivity	75.0%	80.0%	70.0%
Specificity	93.0%	88.4%	93.0%
Positive predictive value	83.3%	76.2%	82.4%
Negative predictive value	88.9%	90.5%	87.0%
Kappa value	0.699	0.675	0.657
*P*	< 0.001	< 0.001	< 0.001
**ADR8**			
Sensitivity	75.0%	75.0%	70.0%
Specificity	95.3%	93.0%	93.0%
Positive predictive value	88.2%	83.3%	82.4%
Negative predictive value	89.1%	88.9%	87.0%
Kappa value	0.733	0.699	0.657
*P*	< 0.001	< 0.001	< 0.001
ADR1: Fp1-F3/Fp2-F4	ADR5: C3-P3/C4-P4	T_1_: After clamping 30 s	
ADR2: F3-C3/F4-C3	ADR6: P3-O1/P4-O2	T_2_: After clamping 1 min	
ADR3: Fp1-F7/Fp2-F8	ADR7: T3-T5/T4-T6	T_3_: After clamping 2 min	
ADR4: F7-T3/F8-T4	ADR8: T5-O1/T6-O2		

### Neurological examination

TCD, RA, and ADR of all patients returned to baseline after carotid artery declamping. All patients underwent physical examination immediately following the operation, and no symptoms of neurological impairment appeared.

## Discussions

Stroke is a severe neurological disease and the leading disabling and fatal disease in adults. At present, the treatment methods of ischemic stroke mainly include drug conservative treatment, interventional treatment, ischemic preconditioning, and surgical treatment (16). CEA is a reliable method for treating and preventing ischemic stroke caused by carotid artery stenosis. It can benefit both symptomatic and asymptomatic patients, but the surgery also risks causing stroke. Shunt surgery, using a silicone tube or shunt to create a bypass of blood flow temporarily, reduces the risk of perioperative ischemic stroke. However, the arterial wall damage caused by shunt surgery increases the risk of carotid artery re-stenosis early after surgery. Therefore, whether to perform shunt during operation is controversial at present. Some scholars recommend routine shunt surgery to avoid the risk of cerebral ischemia, while others recommend selective shunt surgery based on intraoperative monitoring results (17, 18). At present, there is no accepted, utterly reliable method of intraoperative monitoring. Currently, TCD, EEG, somatosensory evoked potential (SEP) and near infrared spectroscopy (NIRS) are the main monitoring methods used in clinical practice.

TCD monitoring is a non-invasive, convenient, and economical method that can continuously monitor the blood flow velocity of large intracranial vessels. It can also be combined with other monitoring technologies to provide suggestions for the intraoperative shunt. In addition, studies have shown that microemboli signals detected by TCD can predict the risk of stroke during and after CEA (19). In conclusion, TCD is a relatively reliable monitoring method for vascular surgery in neurosurgery (19–21).

EEG is a waveform generated by amplifying and recording spontaneous bioelectrical activity on the scalp using sophisticated electronic instruments. Like electrocardiograms, these are the simultaneous electrical activity of millions of cells in the body registered on the body's surface. The electric field generated by these ionic currents can reflect the functional state of the source, and the most relevant is cerebral blood flow. Therefore, EEG has good sensitivity in the early detection of cerebral ischemia and has been used by many centers for CEA IOM (22–24). Cho et al. study, which included one hundred seventy two patients undergoing CEA, found that intraoperative monitoring with EEG reduced overall mortality and significant neurological complications from 2.3% to 1.1% compared with no EEG monitoring (25). Plestis et al. divided 902 patients who underwent CEA into the EEG and non-EEG monitoring groups. They found that EEG monitoring reduced the risk of perioperative stroke from 2.19% (13 cases) to 0.32% (1 case) and EEG could predict the occurrence of stroke during surgery (26). Similarly, Salvian et al. found that in 305 CEA procedures, EEG monitoring to determine whether or not to perform shunt surgery reduced the incidence of perioperative stroke from 4.4%−0.5%(27). However, the complexity of EEG images and the need for accurate interpretation by trained professionals limit the use of EEG to some extent. A new type of processed electroencephalogram, called QEEG, was developed to allow clinicians to interpret the results. Many studies have confirmed that QEEG, similar to EEG, has high sensitivity in detecting cerebral ischemia, especially in the changes of fast rhythm α wave and slow rhythm δ wave (13, 28). Therefore, two QEEG indicators, RA and ADR, were selected for our study. Considering the advantages of TCD in real-time monitoring of middle cerebral artery blood flow velocity and the comprehensive level of vascular ultrasound department in our institution, we chose TCD as the reference standard for intraoperative monitoring.

In this study, twenty patients had V-MCA decline below 50% of baseline after carotid artery clipping, seven patients had an intraoperative shunt, and only two of these seven patients had severe contralateral carotid stenosis. Preoperative digital subtraction angiography (DSA) reports showed that the Willis circles of these seven patients were not developed and the collateral circulations were poor, which could explain why the other 5 patients without severe contralateral carotid artery stenosis needed shunt surgery. In this study, the time for the successful placement of shunt tubes during surgery was about 3–5 min. Therefore, several time points before successful placement of shunt tubes were selected for data analysis to avoid the influence of shunt on grouping during statistical analysis. They are 30 s, 1 min, and 2 min after carotid artery clamping. In addition, the V-MCA improved substantially or returned to near baseline levels after successful shunt placement, which did not meet the grouping criteria for the ischemic group.

EEG is susceptible to factors such as original chronic brain infarction, depth of anesthesia and electrolytes, which may affect the results. In this study, all patients had no sequelae of stroke that affecting daily life before surgery, and NIHSS score was below 2 (Table 1). In terms of depth of anesthesia, all patients were managed by the same anesthesiologist to maintain BIS values between 40 and 60. In fact, the intraoperative BIS values were almost always above 50 to minimize the impact of anesthesia on EEG. All patients underwent intraoperative arterial blood gas analysis to maintain appropriate electrolyte levels and PaCO_2_ levels.

Studies have shown that EEG changes such as attenuation of high-frequency activity (such as alpha) and the presence or increase of delta activity are associated with cerebral ischemia (23). In this study, RA and ADR of all electrode channels on the clamping side of all patients in the ischemic group decreased to varying degrees after carotid artery clamping, which was also similar to the results of existing studies (14, 23, 29). For RA, the optimal cut-off distribution of the eight pairs of electrode channels on the clamping side of the internal carotid artery at three-time points decreased by 11% to 16% compared to baseline. Compared with previous results, this threshold appears to be too strict for monitoring cerebral ischemia, which may be due to the low baseline setting and the influence of anesthetics. All patients in this study received total intravenous anesthesia, strictly following a standardized anesthesia procedure. After induction of anesthesia and the patient's overall conditions were stable, TCD monitoring and blood pressure baseline were set before internal carotid clamp, during which time the patient's EEG may have changed. It is well-known that many anesthetic drugs, such as propofol, have protective effects on the brain, reducing cerebral blood flow and metabolic rate. Studies have shown that using propofol to maintain anesthesia can cause electroencephalograms to switch from high-frequency, low amplitude oscillations to low-frequency oscillations (30).

As shown from Tables 4, 5, RA values of different electrode channels at different time points on the carotid artery clamping side had 75–90% sensitivity, 76.7–93% specificity, 64.3–81.8% positive predictive value, and 88.6–95.7% negative predictive value in predicting cerebral ischemia. The sensitivity, specificity, positive predictive value, and negative predictive value of ADR of different electrode channels at different time points for predicting cerebral ischemia ranged from 65 to 85%, 81.4 to 97.7%, 65.2 to 93.3%, and 85.1 to 92.1%, respectively. There were twenty patients whose V-MCA decreased by more than 50% during carotid artery clamping. Among these twenty patients, the RA values of sixteen to eighteen patients decreased by more than the optimal cut-off point at different time points of different electrode channels and the ADR values of different electrode channels at different time points decreased beyond the optimal cut-off point in 12 to 17 patients. There are two possible reasons for this false negative. On the one hand, TCD measures blood flow velocity in the middle cerebral artery, while EEG measures bioelectrical activity across the entire brain region. These two methods are fundamentally different. On the other hand, analyzing the TCD values of these false-negative cases, it was found that the decline in V-MCA in some patients was at the critical value of TCD monitoring, that is, a 52% or 53% decrease compared with the baseline. This critical blood flow decline may not cause a significant decrease in bioelectric activity at the cellular level, that is, RA and ADR will not change to reach the ischemic threshold. Among the 43 patients whose V-MCA decreased by < 50%, 4–9 patients (13 patients with RA4 channel at 2 min) were observed whose RA values at different time points decreased beyond the optimal cut-off point. The ADR values of different electrode channels at different time points decreased more than the optimal cut-off point value of 2–8 patients. At the time point 2 min, the false positive rate of the RA4 channel was higher, which may be caused by the electrode artifact caused by accidentally touching the electrode cap during operation. The possible reason for the false positive of the whole non-ischemic group is that the compensatory capacity of cerebral blood flow in these patients has approached or reached the compensatory limit, and the brain tissue on the stenosis side can no longer tolerate the decrease of cerebral blood flow. In other words, even a slight decline in cerebral blood flow can cause a decrease in cellular metabolism, leading to changes in QEEG.

We also made a four-grid table for grouping according to TCD and the optimal cut-off point value of QEEG. We calculated the Kappa values of different electrode channels at different time points. The Kappa values of RA ranged from 0.583 to 0.786, and those of ADR ranged from 0.542 to 0.733. This indicates that the QEEG used in this study has a good consistency with TCD in monitoring cerebral ischemia.

Although the primary objective of our study was not to demonstrate the ability of QEEG to predict postoperative neurological complications, neurophysical examinations were performed after surgery. The results showed that no patients developed neurological deficits immediately after surgery, which may be related to the strict threshold of TCD we selected.

The advantage of this study is that it is a prospective study, ensuring the reliability of data collection, using standardized anesthesia management procedures for all patients, and performed by the same group of surgeons.

The deficiencies of this study are as follows. First of all, the patients recruited to this study were all men. The patients' electrode cap of QEEG monitoring need to be placed on the scalp, in order to ensure the acquired data is reliable. This requires that the patient's hair be as short as possible to ensure good contact with the scalp electrode cap. In contrast, female patients, because of the length of the hair and the demand for humanistic care, may cause gender bias. The results of the study may not apply to all populations. Secondly, this study was a single-center study with small sample size and limited the universality of the study due to specific surgical modalities that may result in biased results. In order to check whether the cut-off points obtained in the RA and ADR of this sample are extrapolable to other populations, they should be externally validated. Thirdly, blood pressure and cerebrovascular diameter can affect cerebral blood flow. In this study, blood pressure of all patients was increased by up to 20% after clamping compared with baseline blood pressure, but cerebrovascular diameter was not considered, which may cause potential bias. Furthermore, we did not conduct imaging studies, such as MRI, were used to determine symptomatology or to obtain information about parenchymal lesions prior to surgery.

## Conclusion

Our study demonstrated that QEEG is a promising monitoring method undergoing CEA under general anesthesia and has good consistency with TCD.

## Data availability statement

The raw data supporting the conclusions of this article will be made available by the authors, without undue reservation.

## Ethics statement

The studies involving human participants were reviewed and approved by the Institutional Ethics Board of Xuanwu Hospital Capital Medical University. The patients/participants provided their written informed consent to participate in this study. Written informed consent was obtained from the individual(s) for the publication of any potentially identifiable images or data included in this article.

## Author contributions

Study design and manuscript preparation: GZ, GF, and TW. Data acquisition: GZ, LZ, SF, and YA. Statistical analysis and data management: GZ and CK. Manuscript editing and review: TW. All authors contributed to the article and approved the submitted version.
